# Characterization of *Salmonella* Occurring at High Prevalence in a Population of the Land Iguana *Conolophus subcristatus* in Galápagos Islands, Ecuador

**DOI:** 10.1371/journal.pone.0023147

**Published:** 2011-08-10

**Authors:** Alessia Franco, Rene S. Hendriksen, Serena Lorenzetti, Roberta Onorati, Gabriele Gentile, Giacomo Dell'Omo, Frank M. Aarestrup, Antonio Battisti

**Affiliations:** 1 Istituto Zooprofilattico Sperimentale delle Regioni Lazio e Toscana, Rome, Italy; 2 National Food Institute (DTU-Food), Technical University of Denmark, Kongens Lyngby, Denmark; 3 Dipartimento di Biologia, Università degli Studi di Roma “Tor Vergata”, Rome, Italy; 4 Ornis italica, Rome, Italy; University of Osnabrueck, Germany

## Abstract

The aim of the study was to elucidate the association between the zoonotic pathogen *Salmonella* and a population of land iguana, *Colonophus subcristatus*, endemic to Galápagos Islands in Ecuador. We assessed the presence of *Salmonella* subspecies and serovars and estimated the prevalence of the pathogen in that population. Additionally, we investigated the genetic relatedness among isolates and serovars utilising pulsed field gel electrophoresis (PFGE) on *Xba*I-digested DNA and determined the antimicrobial susceptibility to a panel of antimicrobials. The study was carried out by sampling cloacal swabs from animals (n = 63) in their natural environment on in the island of Santa Cruz. A high prevalence (62/63, 98.4%) was observed with heterogeneity of *Salmonella* subspecies and serovars, all known to be associated with reptiles and with reptile-associated salomonellosis in humans. Serotyping revealed 14 different serovars among four *Salmonella enterica* subspecies: *S. enterica* subsp. *enterica* (n = 48), *S. enterica* subsp. *salamae* (n = 2), *S. enterica* subsp. *diarizonae* (n = 1), and *S. enterica* subsp. *houtenae* (n = 7). Four serovars were predominant: *S.* Poona (n = 18), *S.* Pomona (n = 10), *S.* Abaetetuba (n = 8), and *S.*Newport (n = 5). The *S.* Poona isolates revealed nine unique *Xba*I PFGE patterns, with 15 isolates showing a similarity of 70%. Nine *S.* Pomona isolates had a similarity of 84%. One main cluster with seven (88%) indistinguishable isolates of *S.* Abaetetuba was observed. All the *Salmonella* isolates were pan-susceptible to antimicrobials representative of the most relevant therapeutic classes. The high prevalence and absence of clinical signs suggest a natural interaction of the different Salmonella serovars with the host species. The interaction may have been established before any possible exposure of the iguanas and the biocenosis to direct or indirect environmental factors influenced by the use of antimicrobials in agriculture, in human medicine or in veterinary medicine.

## Introduction

*Salmonella* is known to be associated with free-living and captive reptiles [1]–[4], and sometimes has been detected at high prevalence rates, among species of the order *Squamata* [5], including *Iguanidae* [6], [7]. Although many *Salmonella* serovars are probably commensal organisms in reptiles [8], they can be a cause of overt disease, especially in captive reptiles with impaired immune system [9]. Human exposure to reptiles and *Iguanidae* is currently considered a significant risk factor for *Salmonella* infections [10], [11] and is frequently reported causing clinical disease [7], [12].

The aim of the study was to (1) elucidate the association of the zoonotic pathogen *Salmonella spp*. in a population of the endemic land iguana, *Colonophus subcristatus* from Galápagos Islands in Ecuador. Additionally, (2) we assessed the presence of *Salmonella* subspecies and serovars and estimated the prevalence of the pathogen in that population of land iguanas. We also (3) investigated the genetic relatedness among isolates and serovars utilising pulsed field gel electrophoresis (PFGE) and (4) determined the antimicrobial susceptibility to a panel of antimicrobials.

## Materials and Methods

### Collection of samples

Cloacal swabs of 63 land iguanas *C. subcristatus* were sampled in December 2003 within their natural environment of the Santa Cruz island, Galápagos in Ecuador. Santa Cruz (986 Km^2^) is the economic and tourism hub of Galápagos, and the island which houses the largest resident human population in the archipelago. Land iguanas, once occurring at several sites, are now limited to the north-western area of the island, partly accessible to tourists, with a population now estimated at 450–500 animals. All samples were collected and exported with the approval of the Galápagos National Park and by the issue of CITES documentation (Galapagos National Park Permit n. 050/03, Export CITES n. 79, Import CITES n. IT/IM/2003/MCE/02328) granted to Gabriele Gentile [13].

### Isolation and identification

The isolation of *Salmonella spp.* was conducted according to a modified protocol of the ISO standard 6579∶2002 [14] using MacConkey agar as the second option of a selective agar and Salmonella-Shigella agar (Oxoid Ltd, Basingstoke, UK) replacing Xylose Lysine Deoxycholate (XLD) agar, both incubated at 37°C for 18–24 hours. The presumptive positive *Salmonella* isolates were subjected to biochemical analysis using the API 20E identification system (bioMérieux, Craponne, France).

### Serotyping

All 63 isolates were initially serotyped at the National Institute of Health, Bangkok, Thailand using slide agglutination with hyperimmune antisera (S & A reagents lab, Ltd, Bangkok, Thailand) characterizing the O and H antigens. The serotype of eight of the isolates; #7b, #28, #54, #56, #59, #60, #62, and #63 were re-confirmed at the DTU-Food, Copenhagen, Denmark using antisera (Staten Serum Institute, Copenhagen, Denmark). The serotypes were assigned according to the Kauffmann-White scheme [15].

### Antimicrobial susceptibility testing

Broth micro-dilution susceptibility testing was performed on all *Salmonella* isolates in 96-well microtitre plates (Trek Diagnostic Systems, Westlake, OH, USA) and interpreted according to the European Committee on Antibiotic Susceptibility Testing (EUCAST) epidemiological cut-offs (www.eucast.org). The following drugs, representative of the most relevant antimicrobial classes active against *Enterobacteriaceae*, were tested: ampicillin, cefotaxime, ceftazidime, ciprofloxacin, chloramphenicol, colistin, florfenicol, gentamicin, kanamycin, nalidixic acid, streptomycin, sulphonamides, tetracycline, and trimethoprim.

### Pulsed field gel electrophoresis (PFGE)

All of the isolates were analyzed for genetic relatedness by PFGE using *Xba*I and according to the United States CDC PulseNet protocol [16]. Electrophoresis was performed with a CHEF-DR III System (Bio-Rad Laboratories, Hercules, California) using 1% SeaKem Gold agarose in 0.5× Tris-borate-EDTA at 6 V with an angle of 120°. Running conditions consisted of one phase from 2.2 to 63.8 s at a run time of 20 h.

## Results

### Prevalence

Sixty-two out of 63 individual samples were positive for *Salmonella spp.*, with an overall prevalence of 98.4% (95% Confidence Interval 92.8–99.9%).

### Serotyping

**Figure 1:**
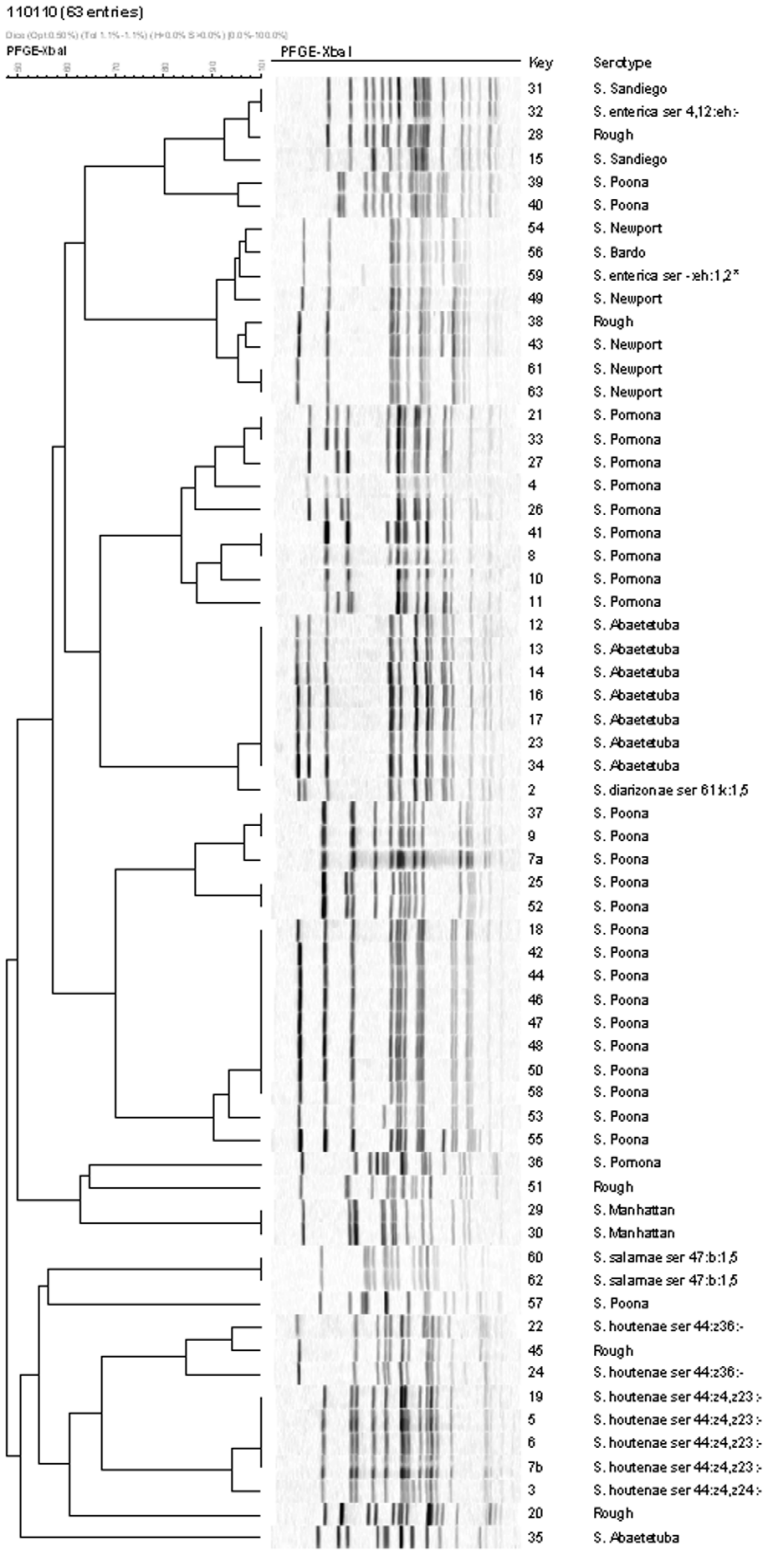
Dendrographic analysis of PFGE (*Xba*I-digested DNA) of *Salmonella spp.* from land iguanas (*C. subcristatus*) sampled in December 2003 from the island of Santa Cruz, Galápagos, Ecuador. *: Somatic phase damaged.

A total of 63 *Salmonella* isolates were obtained from the 62 *Salmonella* positive animals, since one individual sample yielded isolates belonging to two different serovars. Serotyping revealed 14 different serovars among four *Salmonella enterica* subspecies; *S. enterica* subsp. *enterica* (n = 48, 76%), *S. enterica* subsp. *salamae* (n = 2, 3%), *S. enterica* subsp. *diarizonae* (n = 1, 2%), and *S. enterica* subsp. *houtenae* (n = 7, 11%). Additionally, six (10%) of the isolates were untypable of which five (8%) indicated as “rough”; auto agglutinating with saline and one (2%); #59 indicated as “damaged” (Figure 1). Four serovars dominated with more than two isolates per serovar; *Salmonella* Poona (n = 18, 29%), *Salmonella* Pomona (n = 10, 16%), *Salmonella* Abaetetuba (n = 8, 13%), and *Salmonella* Newport (n = 5, 8%).

### Antimicrobial susceptibility

All of the 63 *Salmonella* isolates included the study were susceptible to all the antimicrobials tested, thus demonstrating a wild-type phenotype towards the major classes of antimicrobials used in human and in animal therapy.

### PFGE typing

The 18 *S.* Poona isolates revealed nine unique PFGE patterns of *Xba*I-digested genomic DNA, of which three clusters with ≥2 indistinguishable isolates (Figure 1). One of the clusters contained eight identical isolates and 15 (83%) of the isolates had a similarity of 70%. Interestingly, three isolates (17%) (#39, #40, and #57) of the 18 *S. Poona* isolates were distanced apart from the main group of *S.* Poona isolates. Nine (90%) out of ten *S.* Pomona isolates had a similarity of 84% with two clusters of ≥2 identical isolates. One *S.* Pomona isolate was located apart from the main group of *S.* Pomona isolates. One cluster containing seven (88%) indistinguishable isolates out of the eight *S.* Abaetetuba was observed. The seven *S.* Abaetetuba (I 11:k:1,5) isolates had a similarity of 94% but also grouped with one *S. enterica* subsp. *diarizonae* 61:k:1,5 isolate. One cluster of an identical pattern contained two different serovars; *S.* Sandiego (I 4,[5],12:e,h:e,n,z15) and *S. enterica* subsp. *enterica* 4,12:e,h:-. Equally, four *S. enterica* subsp. *houtenae* 44:z4,z23:- had a similarity of 96% with one *S. enterica* subsp. *houtenae* 44:z4,z24:-. One group of isolates had a similarity of 94% containing four different serovars; *S.* Bardo (I 8:e,h:1,2), *S.* Newport (I 6,8,20:e,h:1,2) and *S. enterica* subsp. *enterica* 43:e,h:1,2. Additionally, clusters of the following serovars; *S. enterica* subsp. *houtenae* 44:z4,z23:- (n = 4), *S.* Newport (n = 2), S. Manhattan (n = 2), and *S. enterica* subsp. *salamae* 47:b:1,5 (n = 2), were observed containing ≥2 indistinguishable isolates. (Figure 1).

## Discussion

The *Salmonella* isolates detected in what is considered a natural land iguana population revealed a wide spectrum of subspecies and serovars. These serovars may be considered as part of their normal bacterial intestinal flora, as none of the animals showed any apparent sign of intestinal or systemic disease. Although the land iguana population on Santa Cruz island does not reach the densities occurring on other islands such as Santa Fé and Plaza Sur, almost all animals harboured *Salmonella*, at a prevalence even higher than reported in land iguanas sampled on these other two minor islands of the Galápagos island chain, in a recent small-scale study [17]. Indeed, most of the animals were colonised by isolates that belong to *S. enterica* subsp. *enterica*, with *S.* Poona, *S.* Pomona, and *S.* Abaetetuba among the most prevalent ones. These serovars, along with *S.* Sandiego and *S.* Manhattan have recently been reported from Galápagos land iguanas sampled on the islands of Santa Fé and Plaza Sur [17]. *S.* Poona and S. Pomona have been reported from other poikilotherm species such as chelonians, iguanas, and lizards; all belonging to the Order Squamata [7], [18], [19]. *S.* Newport is also known to be associated with wild reptiles and amphibians from North America [2], and was detected even in reptiles within supposedly pristine environments; such as wild water chelonians from Texas, the United States [20]. Since all of these serovars have also been associated with human salmonellosis [7], [21]–[23], caution should be taken when capturing and handling such animals for scientific purposes. Moreover, *S.* Poona, *S.* Pomona, and *S.* Newport are also known to be associated with other species of homeotherm domestic and wild mammals [24]–[26]. *S.* Abaetetuba, among the most prevalent serovars in the population of land iguanas studied, has recently been described in environmental waters and in the faeces of migrating cranes (*Grus* spp.) sampled in Japan [27]. The high probability that land iguanas carry multi-host pathogenic *Salmonella* serovars may also have further implications when planning translocation of such wild animals in new environments.

The heterogeneity of *Salmonella* subspecies and serovars in land iguanas from Santa Cruz, may also be related to the past history of its population. Indeed, in the middle 1970s feral dogs almost exterminated land iguanas from Santa Cruz [28]. Remnant individuals were translocated into the corrals of the Galápagos National Park, for the purpose of a captive-breeding program, which was conducted in proximity with premises of other reptiles (giant tortoises). Subsequently, after removing feral dogs, founder individuals and their offspring were repatriated in the original area.

In this study, we also reported the isolation of other subspecies known to be reptile-associated, and with zoonotic potential such as S. enterica subsp. diarizonae and S. enterica subsp. houtenae [29]–[32]. Indeed, only few reports of clinical disease caused by these subspecies are described, thus suggesting very low incidence in the community, and often affecting patients with concurrent diseases or impairment of the immune system.

The dendrogram combining the PFGE patterns with the serotype reveal some interesting constatations (Figure 1). Out of the five untypable “rough” isolates, three clustered with a high similarity to fully serotyped isolates. This would indicate that these might be of the same serotype as the ones with which they cluster but simply autoagglutinated when attempting the conventional serotyping.

We also observed one case where an isolate expressing only one flagellar phase. This isolate was designated as a monophasic variant and most likely of the serovar *S.* Sandiego, as the monophasic isolates share an indistinguishable pattern with a *S.* Sandiego strain. The dendrogram revealed a high PFGE similarity between *S.* Newport (I 6,8:e,h:1,2) and *S.* Bardo (I 8:e,h:1,2). This phenomenon have been assigned as a colonial form variation (the variable expression of minor antigens by different single-colony picks from the same strain) which may occur with the expression of the O:6 antigen by some serogroup C2 serovars [33]. In a recent proficiency test conducted by the World Health Organisation, the organizers allowed for colonial form variations why they did not distinguish between *S.* Newport and *S.* Bardo [34].

In addition, the dendrogram also showed a high PFGE similarity between isolates expressing the same flagellin phases but different somatic phases as between the isolate displaying a damaged somatic phase; I -:e,h:1,2 and *S.* Newport/*S.* Bardo. This observation is interesting as science today is moving towards more DNA/sequenced based technologies. In the future, using only DNA/sequenced based methodologies might show as in this case that some isolates of different serovars (according to Kaufmann-White scheme) are more related compared to other isolates of the same serovar.

It was not surprising that all isolates were susceptible to all of the tested classes of antimicrobials. Indeed, the absence of antibiotic selection pressure in this study's environment and among this wild reptile population is likely to have resulted in *Salmonella* isolates with a wild-type phenotype of susceptibility to antimicrobial drugs. Similar results have been found by Thaller and collaborators [35] for *Enterobacteriaceae* from *C. pallidus*, in Santa Fé island, where the combination of environment conditions and limited human impact led the authors to conclude that, in the absence of chronic antibiotic exposure, the diffusion of acquired antibiotic resistance in wildlife is unlikely. Our results would confirm such a conclusion, especially if considering that in the case of Santa Cruz island human-driven contamination and usage of antimicrobials in humans and domestic animals occur, and might be not as limited as in the previous study. The host interaction with wild-type, pan-susceptible *Salmonella* bacteria may have been established before any possible exposure of the iguanas and the Galápagos biocenosis to environmental factors influenced by the use of antimicrobials in agriculture, in human medicine or in veterinary medicine, including possible use in domestic animals living on the island.

In conclusion, this study revealed a high prevalence of Salmonella of wild-type, pan-susceptible phenotype, and a wide heterogeneity of subspecies and serovars in land iguanas living on Santa Cruz island. The high prevalence and absence of clinical signs in the sampled animals are suggestive of a natural interaction between Salmonella different subspecies and serovars and the host species, so that the bacterium may be considered among the microbiota of the land iguana. The heterogeneity observed in Salmonella may be the result of a pattern of host-bacteria interactions occurring among land iguanas and the biocenosis on Santa Cruz which may be more complex than that occurring on minor islands of the Galápagos island chain. In this respect, within-island exposure dynamics and factors like island size and ecosystem, behaviour, habitat use, human activities and habitat modification may play an important role, still to be investigated.
